# REVOLUTA and WRKY53 connect early and late leaf development in *Arabidopsis*

**DOI:** 10.1242/dev.117689

**Published:** 2014-12-15

**Authors:** Yakun Xie, Kerstin Huhn, Ronny Brandt, Maren Potschin, Stefan Bieker, Daniel Straub, Jasmin Doll, Thomas Drechsler, Ulrike Zentgraf, Stephan Wenkel

**Affiliations:** 1 Center for Plant Molecular Biology, University of Tuebingen, Auf der Morgenstelle 32, 72076 Tuebingen, Germany; 2 Copenhagen Plant Science Centre, University of Copenhagen, Thorvaldsensvej 40, Frederiksberg C 1871, Denmark

**Keywords:** REVOLUTA, HD-ZIPIII, WRKY53, Leaf senescence, Hydrogen peroxide signaling

## Abstract

As sessile organisms, plants have to continuously adjust growth and development to ever-changing environmental conditions. At the end of the growing season, annual plants induce leaf senescence to reallocate nutrients and energy-rich substances from the leaves to the maturing seeds. Thus, leaf senescence is a means with which to increase reproductive success and is therefore tightly coupled to the developmental age of the plant. However, senescence can also be induced in response to sub-optimal growth conditions as an exit strategy, which is accompanied by severely reduced yield. Here, we show that class III homeodomain leucine zipper (HD-ZIPIII) transcription factors, which are known to be involved in basic pattern formation, have an additional role in controlling the onset of leaf senescence in *Arabidopsis*. Several potential direct downstream genes of the HD-ZIPIII protein REVOLUTA (REV) have known roles in environment-controlled physiological processes. We report that REV acts as a redox-sensitive transcription factor, and directly and positively regulates the expression of *WRKY53*, a master regulator of age-induced leaf senescence. HD-ZIPIII proteins are required for the full induction of *WRKY53* in response to oxidative stress, and mutations in *HD-ZIPIII* genes strongly delay the onset of senescence. Thus, a crosstalk between early and late stages of leaf development appears to contribute to reproductive success.

## INTRODUCTION

Senescence is the final stage of leaf development and involves the concerted reallocation of nutrients from the leaves to developing parts of the plant, especially fruits and seeds. Thus, leaf senescence has a major impact on yield quantity and quality, e.g. salvaged nitrogen (N) from wheat leaves accounts for up to 90% of the total grain N content (Kichey et al., 2007). In order to minimize loss of nutrients, plants induce leaf senescence in response to endogenous cues such as plant age and altered hormone homeostasis. However, external factors, such as the availability of water or light quality can also induce senescence, referred to as premature senescence (Ballaré, 1999). Although age-induced senescence tends to maximize seed production, premature senescence describes an exit strategy that is induced in response to sub-optimal growth conditions and is often correlated with severely decreased yields.

The onset and progression of leaf senescence is accompanied by immense changes in the leaf transcriptome. It is estimated that about 20% of all genes are altered in expression upon induction of senescence, implying an important role for transcriptional regulators (Balazadeh et al., 2008; Breeze et al., 2011; Buchanan-Wollaston et al., 2005; Zentgraf et al., 2004). NAC and WRKY transcription factors are over-represented in the senescence transcriptome (Guo et al., 2004) and some members of these two transcription factor families have been shown to play central roles in regulating senescence (Balazadeh et al., 2010, 2011; Besseau et al., 2012; Breeze et al., 2011; Miao et al., 2004; Uauy et al., 2006; Ülker et al., 2007; Yang et al., 2011). WRKY proteins are plant-specific transcriptional regulators that contain a DNA-binding domain of ∼60 amino acids. This domain contains a WRKYGQK motif at the N terminus and a zinc-finger structure at the C terminus, and is called the WRKY domain. Diverse processes, such as the response to pathogens or wounding but also leaf senescence, are controlled by WRKY transcription factors (Rushton et al., 2010). WRKY53, a key player in age-induced leaf senescence, regulates a complex network of downstream targets that promote vast physiological changes associated with the reallocation of nutrients and the induction of cell death (Lin and Wu, 2004; Miao et al., 2004). Owing to its important function, *WRKY53* expression, activity and protein stability are tightly controlled (Zentgraf et al., 2010). When leaf senescence is induced, the *WRKY53* locus is activated by histone modifications H3K4me2 and H3K4me3 (Ay et al., 2009; Brusslan et al., 2012), whereas DNA methylation remains low and unchanged (Zentgraf et al., 2010). Several promoter-binding proteins have already been characterized for *WRKY53* regulation, including WRKY53 itself, other WRKYs and the activation domain protein (AD protein), which has some similarity to HPT kinases and works as an activator of *WRKY53* expression (Miao et al., 2008; Potschin et al., 2014). In addition, a mitogen-activated protein kinase kinase kinase (MEKK1) was characterized to bind directly to the DNA of the *WRKY53* promoter. The binding region of MEKK1 appears to be involved in the switch from leaf age-dependent to plant age-dependent expression of *WRKY53* (Hinderhofer and Zentgraf, 2001; Miao and Zentgraf, 2007). MEKK1 can directly phosphorylate the WRKY53 protein, thereby increasing its DNA-binding activity (Miao and Zentgraf, 2007). As almost all WRKY factors contain WRKY factor-binding sites (W-boxes) in their proximal promoter regions, a complex regulatory WRKY network exists. Besides the transcriptional regulation, WRKY53 protein stability is strongly controlled by a HECT E3-ubiquitin ligase (Miao and Zentgraf, 2010). Moreover, gene expression changes are accompanied by hormonal changes. Although the plant hormones cytokinin and auxin act to delay senescence (Kim et al., 2011; Li et al., 2012), ethylene, abscisic acid (ABA), salicylic acid (SA) and jasmonic acid (JA) strongly promote leaf senescence (Li et al., 2012). Besides hormone homeostasis, elevated hydrogen peroxide levels also trigger senescence (Bieker et al., 2012; Smykowski et al., 2010).

Here, we identify REVOLUTA (REV), a transcription factor known to regulate polarity-associated growth processes in embryos, leaves, stems, vasculature and roots (Carlsbecker et al., 2010; McConnell et al., 2001; Smith and Long, 2010), as a direct regulator of *WRKY53* expression. During early leaf development, REV is involved in establishing the dorsoventral axis of leaves by specifying the domain that will later develop into the upper side of the leaf (Byrne, 2006). REV, also known as INTERFASCICULAR FIBERLESS (IFL), has been shown to play multiple roles in meristem organization, leaf polarity set-up and vascular development (Otsuga et al., 2001; Talbert et al., 1995; Zhong and Ye, 1999). Using a ChIP-Seq approach, we identified REV-binding sites in the *WRKY53* promoter and by qRT-PCR demonstrate that REV promotes *WRKY53* expression. Conversely, plants that carry loss-of-function mutations in *REV* and other *HD-ZIPIII* genes show lower levels of *WRKY53* expression, confirming that HD-ZIPIIIs are also required for *WRKY53* expression. By performing a detailed expression analysis using both *REV* and *WRKY53* GUS-reporter lines, we reveal that both genes have partially overlapping patterns of expression. In wild-type plants, *WRKY53* expression is strongly induced in response to hydrogen peroxide. However, in *rev* mutant plants and in transgenic plants with reduced *HD-ZIPIII* activity, this response is significantly dampened. Furthermore, the ability of REV to bind to the *WRKY53* promoter is also dependent on the redox environment and, under oxidative conditions, less binding is observed. In line with the lower *WRKY53* expression levels, *rev* mutant plants are considerably delayed in age-induced leaf senescence, suggesting a role for HD-ZIPIIIs in this physiological process. Taken together, we conclude that REV is a positive regulator of *WRKY53* expression, which influences the onset of leaf senescence in response to changes in the cellular redox state. Obviously, early and late leaf development are tightly linked by transcriptional networks between HD-ZIPIII and WRKY factors, in which disturbed early development is coupled to extended life span of leaves and delayed senescence.

## RESULTS

### REVOLUTA is a positive regulator of *WRKY53* expression, a major factor controlling age-induced leaf senescence

**Fig. 1.**
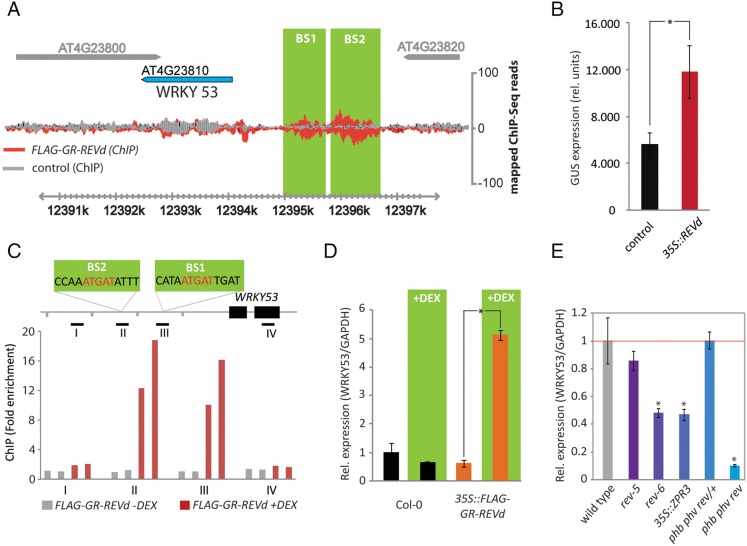
**REVOLUTA binds to the *WRKY53* promoter and is a direct and positive regulator of *WRKY53* expression.** (A) ChIP-Seq results for the binding of REV to the *WRKY53* promoter. Two binding sites (BS) were identified, located −1.3 kb and −2.1 kb upstream of the transcriptional start site. Traces in gray are sequence reads derived from sequencing ChIP DNA from Col-0 wild-type plants; red plots ChIP DNA from dexamethasone-induced *35S::FLAG-GR-REVd* transgenic plants. (B) Transient expression assay in *Arabidopsis* protoplasts. A plasmid with a 2.8 kb *WRKY53* promoter fragment fused to the *GUS* gene was transformed along with a second plasmid containing a *CaMV35S*-promoter (control) or the *CaMV35S*-promoter driving expression of *REVd*. GUS activity was determined ∼15 h after transformation. Data are mean±s.d. *P<0.05. (C) Chromatin-immunoprecipitation qPCR experiments with two biological replicates for *35S::FLAG-GR-REVd* without DEX (gray bars) and *35S::FLAG-GR-REVd* with DEX (red bars) plants testing four positions in the *WRKY53* promoter. Y-axis shows the fold enrichment normalized to the non-induced IPs. Gene map above the chart shows the localization of the REV-binding site identified by ChIP-Seq and the regions that were tested. Distance between two marks along the chromosomes represents 1.0 kb. (D) Real-time quantitative PCR experiments showing expression changes of *WRKY53* in Col-0 (black) and *35S::GR-REVd* (orange) in response to 60 min DEX induction in the presence of the protein biosynthesis inhibitor cycloheximide (CHX). Data are mean±s.d. *P<0.05. (E) Expression of *WRKY53* was analyzed in different *rev* mutant plants (*rev-5*, *rev-6*, *phb phv rev/+* and *phb phv rev*) and in plants with reduced activity of HD-ZIPIII proteins (*35S::ZPR3*). The bars indicate expression levels relative to wild type, including standard errors of the mean of three individual biological experiments. *P<0.05.

REVOLUTA is a member of the class III homeodomain leucine zipper (HD-ZIPIII) transcription factor family that regulates various polarity-associated growth processes during development (Carlsbecker et al., 2010; McConnell et al., 2001; Smith and Long, 2010), but plays an additional role in shade-induced growth promotion (Bou-Torrent et al., 2012; Brandt et al., 2012). REVOLUTA expression is controlled by the microRNAs *miR165* and *miR166* at the post-transcriptional level (Rhoades et al., 2002), and by the association with small leucine-zipper-type microProteins at the post-translational level (Kim et al., 2008; Staudt and Wenkel, 2011; Wenkel et al., 2007). Using a genome-wide chromatin-immunoprecipitation sequencing approach (ChIP-Seq), we recently identified binding regions for REV across the *Arabidopsis* genome (Brandt et al., 2012). This analysis revealed binding of REV to the promoter of the *WRKY53* transcription factor (Fig. 1A). Transient promoter-GUS experiments in *Arabidopsis* protoplasts revealed an induction of *WRKY53* expression after co-transformation of *35S::REVd*, a dominant microRNA-resistant version of REV (Fig. 1B). Quantitative ChIP-PCRs confirmed the binding of REV to the ChIP-Seq identified binding motifs (Fig. 1C). For better control of REV activity, we constructed transgenic plants expressing REVd fused to the rat glucocorticoid receptor carrying an N terminal FLAG epitope. In response to dexamethasone (DEX) induction, the chimeric FLAG-GR-REVd fusion protein translocates to the nucleus, where it can associate with DNA and alter the expression of target genes. In response to DEX induction, REV can significantly upregulate *WRKY53* expression (Fig. 1D), while seedlings carrying mutations in *REV* and plants with globally reduced HD-ZIPIII activity show reduced levels of *WRKY53* mRNA (Fig. 1E), thus supporting a new role for REV as a direct and positive regulator of *WRKY53*.

### *REVOLUTA* and *WRKY53* have overlapping patterns of expression

**Fig. 2.**
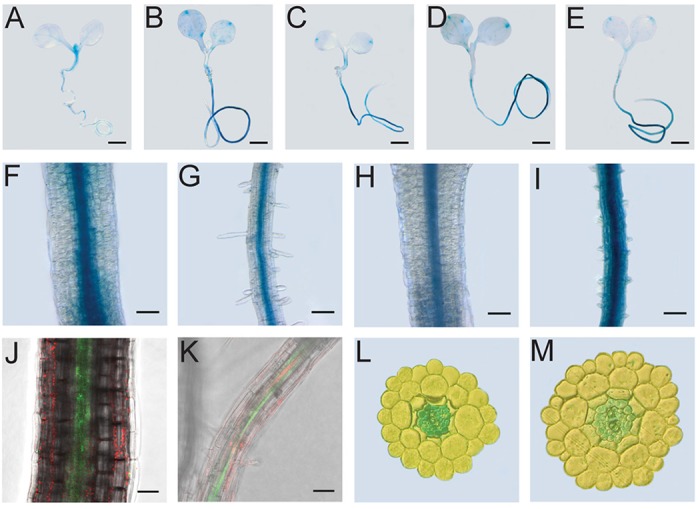
**Expression analysis of *REV* and *WRKY53*.** (A-I) Spatial patterns of expression of *REV* (A,F,G) and *WRKY53* (B-E,H,I) in 8-day-old *Arabidopsis* seedlings. GUS staining of *REV::GUS* (A), *WRKY53::GUS* (B) in the Col-0 ecotype and *WRKY53::GUS, rev5* (C), *WRKY53::GUS, 35S::miR165* (D), *WRKY53::GUS, 35S::ZPR3* (E) seedlings. Scale bars: 1 mm. (F-I) Hypocotyls (F,H) and roots (G,I). (J,K) The pattern of GFP accumulation in the hypocotyl (J) and root (K) vascular tissue of 8-day-old plants carrying the *REV::REV-GFP* transgene. Scale bars: 50 µm. (L,M) Cross-sections of roots of 10-day-old seedlings reveal *REV* (L) and *WRKY53* (M) expression in the vascular cylinder.

*REVOLUTA*, as well as the other class III HD-ZIP transcription factors of *Arabidopsis*, have a distinct expression pattern, confining their expression to the adaxial domain of developing leaves, the xylem part of the vasculature, the pro-vasculature and the shoot apical meristem. Both *WRKY53* and *REV* are expressed in young seedlings (Fig. 2A,B). Even though REV function was initially described for polarity-associated growth processes during early leaf development, REV is still expressed at later stages of development (supplementary material Fig. S1) and an additional function in shade avoidance has recently been assigned to REV (Brandt et al., 2012). In comparison with the vascular expression pattern of *REV*, *WRKY53* shows a broader less-specific pattern of expression and is most highly expressed in old leaves (Miao and Zentgraf, 2007). In genetic backgrounds with reduced *REV* mRNA [*rev-5* (Fig. 2C), *35S::miR165a* (Fig. 2D)] or with reduced REV protein activity (*35S::ZPR3*; Fig. 2E), the spatial expression of *WRKY53* is more restricted to hydatodes and overall expression levels appear to be much lower in leaf tissue. In older seedlings, expression of both genes is found in vascular strands (Fig. 2F-M). Surprisingly, high co-expression is observed in the root vasculature at all investigated stages of development. It is not known whether WRKY53 has an additional function in root development but it might be important to note that the expression in the root vascular appears to be independent of HD-ZIPIII function (Fig. 2B-E).

Using publicly available microarray data (http://bar.utoronto.ca), we also analyzed at which stages of development and in response to which treatments *REV* and *WRKY53* are co-expressed (supplementary material Fig. S2). We find evidence for co-expression during early developmental stages but not during the later stages of leaf development. This discrepancy suggests that *REV* mRNA is not upregulated at late stages of leaf development but residual protein could respond to a cellular signal and induce the expression of REV-regulated senescence targets. However, our GUS expression analyses using *REV::GUS* plants indicate that REV is still expressed to certain extends in older leaves (supplementary material Fig. S1).

In order to identify other direct REV targets that show an expression pattern resembling *WRKY53*, we surveyed recently published timecourse microarray datasets (Reinhart et al., 2013) that revealed 119 genes to be upregulated in response to REVOLUTA induction. Our ChIP-Seq datasets resulted in the identification of 286 high confidence REV-binding sites (corresponding to 552 potentially regulated genes) across the entire *Arabidopsis* genome (Brandt et al., 2012). By comparing both datasets, we could identify 18 of the 119 REV-regulated genes (15% of the REV upregulated set) to have REV-binding sites in their respective promoters (Table 1). *WRKY53* is among these 18 genes and we investigated whether other senescence-related genes could be identified in this dataset. A genome-wide survey with a high temporal resolution classified thousands of genes as differentially expressed senescence genes (DESGs) (Breeze et al., 2011). Interestingly, REV was also classified as a DESG, showing a dip of expression at the onset of leaf senescence. Furthermore, nine out of the 18 potential direct REV targets (Table 1) were also classified as DESGs, implying that REV might have an additional function in late developmental stages.

**Table 1.**
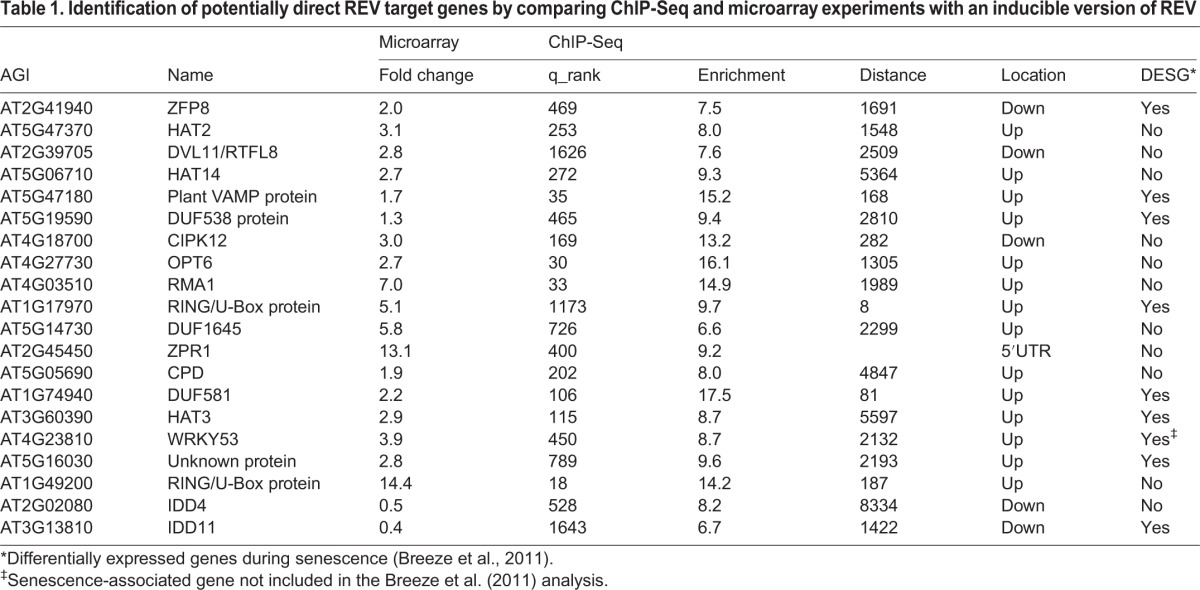
Identification of potentially direct REV target genes by comparing ChIP-Seq and microarray experiments with an inducible version of REV

### *WRKY53* expression is modulated in response to oxidative stress in a REVOLUTA-dependent manner

**Fig. 3:**
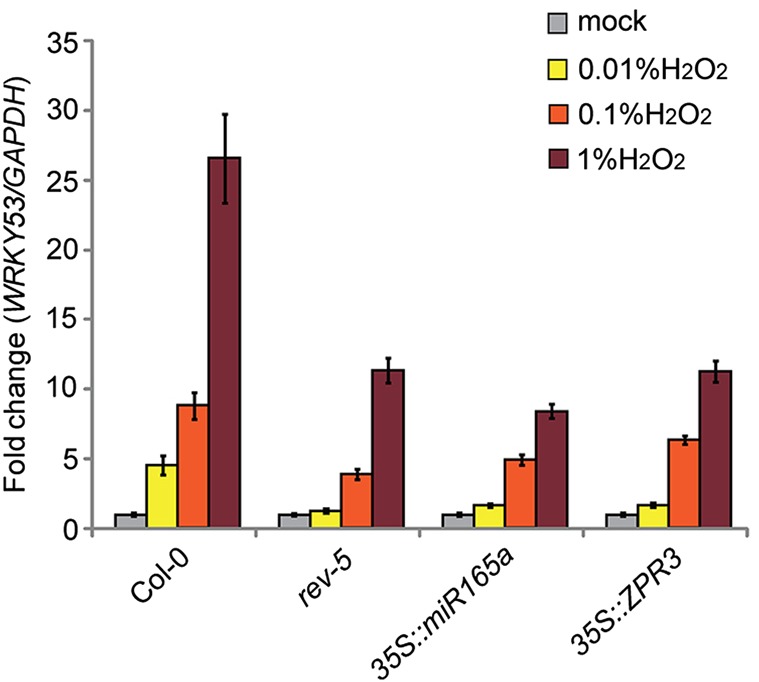
**HD-ZIPIII activity is required for H_2_O_2_-mediated upregulation of *WRKY53*.** Real-time qPCR experiment showing *WRKY53* induction in response to hydrogen peroxide treatment in wild-type and *rev* mutant plants. Three-week-old plants were treated with different concentrations of H_2_O_2_ [0% (mock; gray bars), 0.01% (yellow bars), 0.1% (orange bars) and 1% (red bars)] for 40 min. Data are representative relative expression changes (fold change) of the mean of four technical replicates±s.d. Similar expression changes have been observed in at least two independent biological experiments.

*WRKY53* expression is strongly upregulated in response to hydrogen peroxide as part of the age-induced senescence-promotion pathway (Miao et al., 2004). Because REV is a novel upstream regulator of *WRKY53* expression and possesses a domain that is suggestive of sensing changes in the redox state of the cell, we investigated whether REV is required for the induction of *WRKY53* expression in response to oxidative stress. Therefore, we grew Col-0 wild-type plants and mutant plants with reduced HD-ZIPIII activity (*rev5*, *35S::miR165a* and *35S::ZPR3*) on soil for 3 weeks in long-day conditions. In order to elicit oxidative stress, plants were sprayed with hydrogen peroxide solutions of different concentrations (0.01%, 0.1% and 1%) and plant material was harvested before and after spraying. Subsequent RNA isolation, cDNA synthesis and quantitative PCR analysis revealed a strong induction of *WRKY53* in response to H_2_O_2_ application in Col-0 wild-type plants. These changes of *WRKY53* mRNA levels were significantly dampened in *rev* mutant plants (*rev-5*) and *35S::miR165a*, and in plants with reduced HD-ZIPIII activity (*35S::ZPR3*), indicating that REV activity is required for high-level *WRKY53* induction in response to oxidative stress signaling (Fig. 3). To assess which externally applied hydrogen peroxide concentration is able to elicit redox changes that would occur under natural conditions, we measured intracellular hydrogen peroxide levels after applying heat stress and compared them with the intracellular levels reached after external application of H_2_O_2_ by spraying. To be sure that only intracellular H_2_O_2_ is measured, we used non-fluorescent H_2_DCFDA (2′,7′-dichlorodihydrofluorescein diacetate), which is converted to the highly fluorescent 2′,7′-dichlorofluorescein upon cleavage of the acetate groups by intracellular esterases and subsequent oxidation. The increase in intracellular H_2_O_2_ was similar 1 h after heat treatment and 1 h after spraying 0.1% H_2_O_2_ but dropped more rapidly in the H_2_O_2_-treated samples. This indicates that external application of 0.1% H_2_O_2_ leads to intracellular changes in the range of an oxidative burst in stress response (supplementary material Fig. S3).

### REVOLUTA is a redox-sensitive transcription factor

REV is a positive regulator of *WRKY53* expression and is required for high level of *WRKY53* induction in response to oxidative stress. This could be either due to an upregulation of *REV* mRNA in response to oxidative stress or to a response of the REV protein to altered redox conditions. To test whether *REV* mRNA is upregulated in response to hydrogen peroxide treatment, we treated Col-0 wild-type plants with H_2_O_2_ and performed quantitative RT-PCRs. We detected no induction of *REV* mRNA but a slight decrease in response to high levels of hydrogen peroxide (supplementary material Fig. S4), excluding the idea that *REV* is transcriptionally upregulated in response to oxidative stress.

**Fig. 4.**
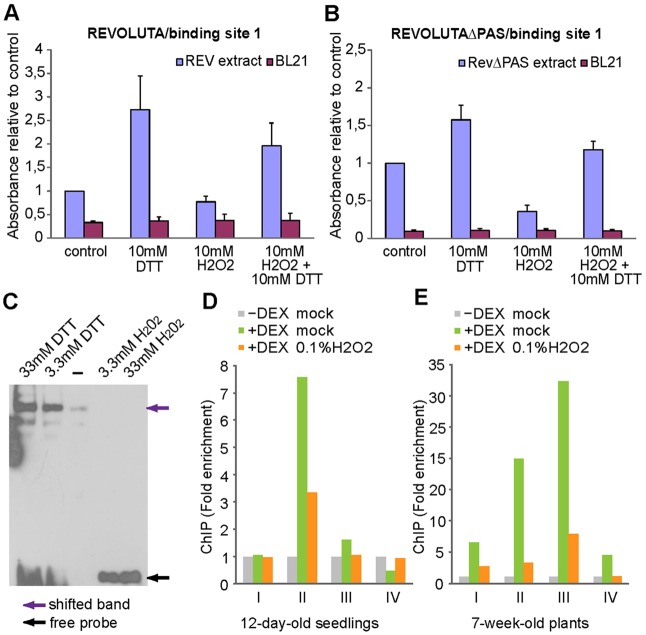
**Redox-mediated regulation of REVOLUTA-DNA-binding capability and influence of the PAS domain.** (A,B) Redox-DPI-ELISAs. The DNA-protein interaction assays were performed by using 5′ biotinylated complementary annealed oligonucleotides coupled to a streptavidin-coated ELISA plate. Crude *E. coli* extracts (25 µg) expressing recombinant REV or REVΔPAS were pre-incubated with different concentrations of DTT and H_2_O_2_ to examine a redox state-dependent binding of REV. In order to test the reversibility of the redox effect, high concentrations of H_2_O_2_ were added first and then oxidizing conditions were reversed by addition of DTT. After binding, biotinylated DNA-protein complexes were detected using anti His-HRP conjugated antibodies. Results for REV binding site 1 of the *WRKY53* promoter are shown. *E. coli BL21* cells transformed with the empty vector were used as background control. (C) Non-radioactive electrophoretic mobility shift assays. Purified GST-REV protein was incubated with a biotinylated oligonucleotide containing the HB9-binding motif (Wenkel et al., 2007) in the presence of different redox conditions. After gel electrophoresis and subsequent blotting, the biotinylated DNA probe was detected with a HRP-strepatividin substrate. (D,E) Chromatin-immunoprecipitation qPCR assays of *35S::FLAG-GR-REVd* plants. Twelve-day-old seedlings (D) and 7-week-old transgenic plants (E) were treated with mock substrate (0.1% ethanol), DEX or 0.1% H_2_O_2_ and DEX. H_2_O_2_ was given 15 min prior to 45 min of DEX induction. Fold enrichment for the same primer sets as in Fig. 1 is shown.

It has been shown that proteins of the class II homeodomain leucine-zipper (HD-ZIPII) family from sunflower interact with DNA in a redox-sensitive manner (Tron et al., 2002). To test whether REV shows also redox-dependent DNA binding, we performed redox-sensitive DPI-ELISA experiments. Therefore, crude lysate of *E. coli* cells expressing HIS-tagged REV protein were prepared and incubated with streptavidin plates pre-loaded with biotinylated oligonucleotides containing the REV-binding site 1 of the *WRKY53* promoter (W53-BS1). ELISA plates were then washed and subsequently incubated with HRP-tagged anti-HIS antibodies. Enhanced signal was detected in the control binding reaction (HIS-REV lysate versus a lysate from BL21 cells expressing the empty vector control), indicating that HIS-REV binds to the W53-BS1 element (Fig. 4A). As observed for the sunflower HD-ZIPII proteins (Tron et al., 2002), REV also showed enhanced binding in response to reducing conditions (10 mM DTT), whereas in response to oxidative conditions (10 mM H_2_O_2_) DNA-binding was reduced (Fig. 4A). This negative effect is reversible as the subsequent addition of 10 mM DTT was able to restore REV DNA binding.

We examined the possibility of whether the C-terminal PAS-domain of REV might act as a redox sensor domain. Redox-DPI-ELISA experiments with HIS-REV lacking the PAS-domain (HIS-REVΔPAS) showed the same redox-sensitive behavior as observed for HIS-REV (Fig. 4B). However, without the PAS-domain, REV-DNA binding was strongly enhanced, supporting the idea that the PAS-domain regulates REV activity via a steric masking mechanism, as proposed by Magnani and Barton (2011). It is conceivable that the observed redox effects in the ELISA system are due to an influence of *E. coli* proteins on the activity of REV. To exclude such effects, we purified GST-REV protein from *E. coli* and performed *in vitro* gel retardation assays in the presence of reducing agents (DTT) and oxidizing agents (H_2_O_2_) (Fig. 4C). These gel-shift experiments largely confirm the results obtained by redox-DPI-ELISA and confirm that REV activity can be modulated by the intracellular redox state.

To validate redox-sensitive DNA binding *in planta*, we treated *35S:*:*FLAG-GR-REVd* transgenic plants with either a mock substrate (0.1% ethanol), dexamethasone (DEX) or DEX+0.1% H_2_O_2_. In 12-day-old seedlings, we detected REV binding to binding site 2 (fragment II) and no binding was observed to binding site 1 (fragment III). When treated with hydrogen peroxide prior DEX induction, binding to binding site 2 was significantly affected (Fig. 4D), indicating that REV DNA binding is indeed redox sensitive. The same experiment with 7-week-old plants revealed that, at later developmental stages, both binding sites are occupied by REV and the binding seems to be enhanced but exhibits the same redox sensitivity (Fig. 4E). Taken together, we demonstrate that REV shows a stage-specific redox-dependent DNA-binding behavior and that oxidizing conditions decrease the ability to bind DNA *in vitro* and *in vivo*.

### Mutations in the *REVOLUTA* gene or the overall reduction of HD-ZIPIII activity delay the onset of leaf senescence

**Fig. 5.**
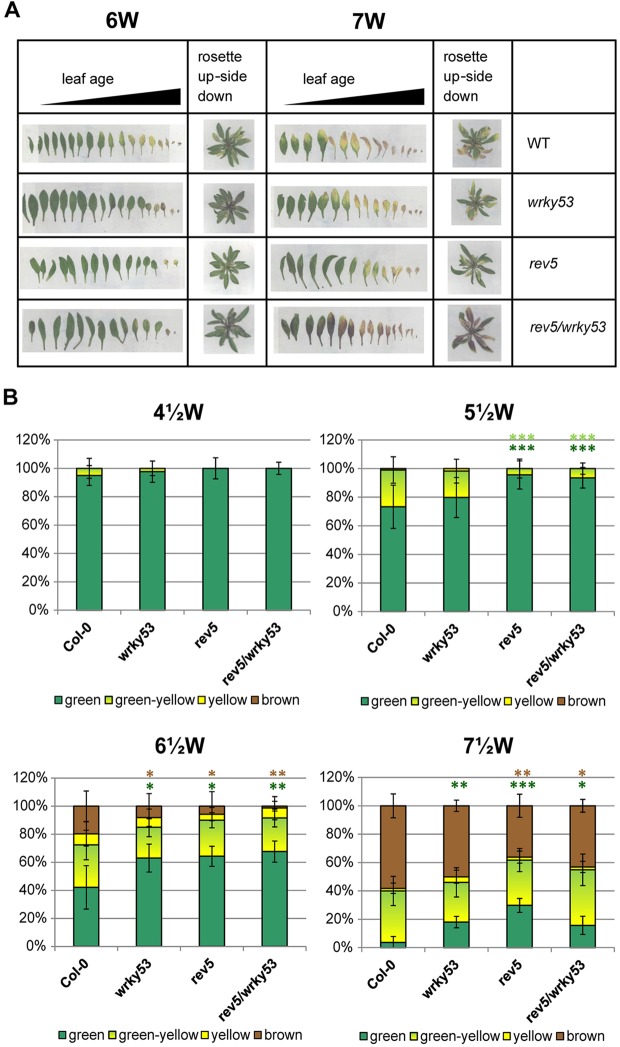
**Genetic interaction of *REV* with *WRKY53*.** (A) Rosette leaves of 6- and 7-week-old representative plants were sorted according to their age; whole rosettes were also photographed upside down to visualize the older leaves. (B) For a quantitative evaluation of leaf senescence, plants were harvested in a weekly rhythm and leaves of at least six plants were categorized into four groups according to their leaf color: (1) ‘green’; (2) leaves starting to become yellow from the tip as ‘yellow-green’; (3) completely yellow leaves as ‘yellow’; and (4) dry and/or brown leaves as ‘brown/dry’. The percentages of each group with respect to total leaf numbers are presented. Error bars indicate s.d. Student's *t*-test was performed comparing leaf counts of *wrky53*, *rev5* and *rev5wrky53* with Col-0 numbers, *P<0.05, **P<0.005, ***P<0.0005. *n*=7-15.

One function of the WRKY53 protein is the regulation of the onset of senescence, documented by the phenotype of the *wrky53* mutant showing delayed senescence. As REV is an activator of *WRKY53* expression, we expected *rev* mutant plants to also display a delayed senescence phenotype. Our analysis revealed that plants carrying mutations in REV or plants with greatly reduced HD-ZIPIII activity are significantly delayed in senescence, while overall development is not retarded, which clearly confirms a role of HD-ZIPIII proteins in this process (Fig. 5; supplementary material Figs S5, S6). Furthermore, the phenotype of *rev5* was even stronger than that of *wrky53*, indicating that *WRKY53* might not be the only senescence-associated gene regulated by REV.

**Fig. 6.**
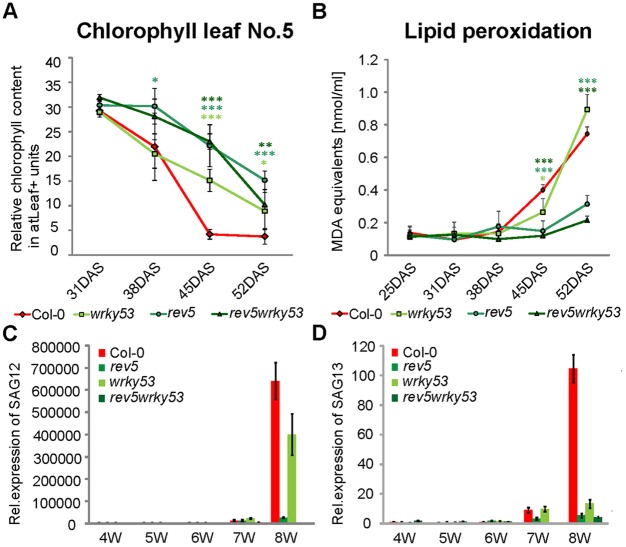
**Molecular senescence parameters.** (A) Chlorophyll contents of number 5 leaves from *Arabidopsis* Col-0, *wrky53*, *rev5* and *rev5wrky53* plants. Left axis indicates atLeaf+ values. Plant age is indicated in days after seeding (DAS). (B) Lipid peroxidation in Col-0, *wrky53*, *rev5* and *rev5wrky53* plants. Values represent mean of at least three biological replicate±s.d. Comparison of means and the determination of statistical differences was carried out using Student's *t*-test (*P<0.05, **P<0.005 and ***P<0.0005). (C,D) qRT-PCR expression analysis of the senescence marker genes *SAG12* and *SAG13*. All values were normalized to *GAPDH* expression. Error bars indicate s.d. of four technical replicates.

Overexpression of the small leucine-zipper-type microProtein ZPR3, which largely reduces the activity of HD-ZIPIIIs, led to a further enhancement of the senescence phenotype, which was ameliorated in the *wrky53* mutant background (supplementary material Fig. S3). This confirms that the senescence phenotype is mediated by deregulation of *WRKY53* expression through HD-ZIPIIIs but also suggests that additional HD-ZIPIIIs are involved, as the senescence phenotype of *35S::ZPR3* plants is much stronger compared with *rev5* mutants (Fig. 5; supplementary material Figs S5,S6). Consistent with the phenotype, two typical senescence-related physiological parameters, the decrease in chlorophyll content and the increase in lipid peroxidation, were also delayed in *wrky53*, *rev5* and *rev5 wrky53* mutants (Fig. 6A,B). Furthermore, the mRNA expression levels of *SENESCENCE ASSOCIATED GENE* 12 (*SAG12*) and *SAG13*, which are commonly used as senescence marker genes, were significantly reduced at the late developmental stages in *wrky53*, *rev5* and *rev5 wrky53* mutants compared with Col-0 wild-type plants (Fig. 6C,D). Taken together, these results confirm that REV acts upstream of *WRKY53* in the control of age-induced senescence.

**Fig. 7.**
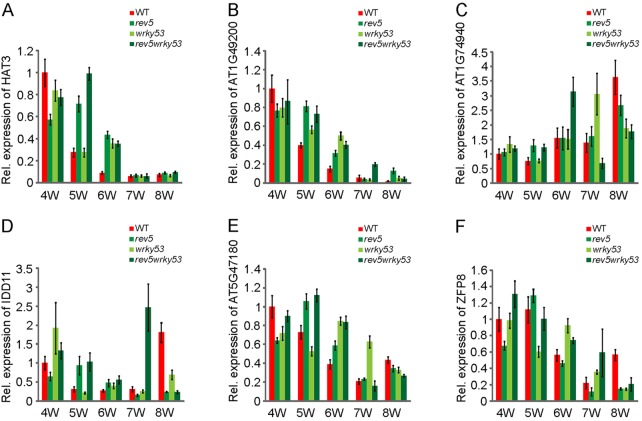
**qRT-PCR of other REV target genes differentially expressed during senescence.** Quantitative real-time PCR profiling of putative REV target genes at late developmental stages in wild-type and mutant plants (4-, 5-, 6-, 7- and 8-week-old plants). (A-F) Expression changes over time of *HAT3*, *AT1G49200*, *AT1G74940*, *IDD11*, *AT5G47180* and *ZFP8*. The Y-axis represents the relative expression level normalized to *GAPDH*. Error bars indicate s.d. of four technical replicates.

Depletion of *REV* delays the onset of leaf senescence more efficiently than depletion of *WRKY53*. To further investigate the possibility that REV acts upstream of several senescence-associated genes, we focused our attention on the potential direct REV targets classified as DESGs (Table 1). Here, we decided to investigate three groups of genes: (1) genes whose expression decreases with age (*HAT3* and *AT1G49200*); (2) genes whose expression increases with age (*AT1G74940* and *IDD11*); and (3) genes whose expression decreases with age but rises during senescence (*AT5G47180* and *ZFP8*). In the first group of genes, we found that expression in *wrky53*, *rev5* and *rev5 wrky53* mutants is maintained at a higher level towards the onset of senescence (weeks 5 and 6), whereas expression levels are dropping rapidly in wild-type plants (Fig. 7A,B). For the second group of genes whose expression increases with age in wild-type plants, we detected elevated levels in *wrky53*, *rev5* and *rev5 wrky53* mutants at early developmental stages (weeks 4 and 5) and decreased levels at the late stages (Fig. 7C,D). Expression of the third group of genes is also altered at various time points in *wrky53*, *rev5* and *rev5 wrky53* mutants compared with Col-0, but in all lines the transcriptional increase during senescence is diminished (Fig. 7E,F), further corroborating the idea that loss of *REV* function profoundly alters the senescence transcriptome, which might be causative for the strong senescence phenotype of *rev* mutant plants.

Loss-of-function *wrky53* mutant plants do not show obvious developmental defects during early leaf development, indicating that WRKY53 is not required for REV function at these stages of development. However, the severe *35S::ZRP3-*induced leaf phenotype is ameliorated in the *wrky53* mutant background, suggesting that the action of other HD-ZIPIIIs involves WRKY53 also at early stages (supplementary material Fig. S7). Nonetheless, WRKY53 protein levels are most likely very low during these early stages of development due to the degradation of WRKY53 by the HECT domain ubiquitin ligase UPL5, which is highly expressed in young leaves (Miao and Zentgraf, 2010). Taken together, we discovered that HD-ZIPIIIs interact with *WRKY53* genetically to promote age-induced leaf senescence, and disruption of early leaf development correlates with delayed senescence and extended life span of leaves.

### Functional analyses of root-specific co-expression patterns of *REV* and *WRKY53*

It is unknown which tissues are involved in the perception of senescence signals and conversion of these into the senescence triggers. We find co-expression of *REV* and *WRKY53* during the early stages of leaf development. Later in development, co-expression was very obvious in the vasculature of the leaves and in the root vascular cylinder (Fig. 2L,M), although both *REV* and *WRKY53* are expressed throughout development (supplementary material Fig. S1). This is in agreement with the finding that REV is involved in the induction of *WRKY53* expression by hydrogen peroxide and that very high levels of hydrogen peroxide were observed in vascular tissue indicated by DAB staining of leaf sections (Zimmermann et al., 2006). Moreover, it remains tempting to speculate that the root might also act as a senescence sensor; however, whether roots play a role during onset and progression of senescence has not yet been determined and whether and to what extent hydrogen peroxide is transported through the vasculature over long distances is also not known so far. Auto-propagating waves of reactive oxygen species (ROS) that rapidly spread from the initial site of exposure to abiotic stress to the entire plant are involved in conferring systemic acquired acclimation, also allowing a much faster transcriptome and metabolome reprogramming of systemic tissues in response to abiotic stress (Mittler et al., 2011; Suzuki et al., 2013).

**Fig. 8.**
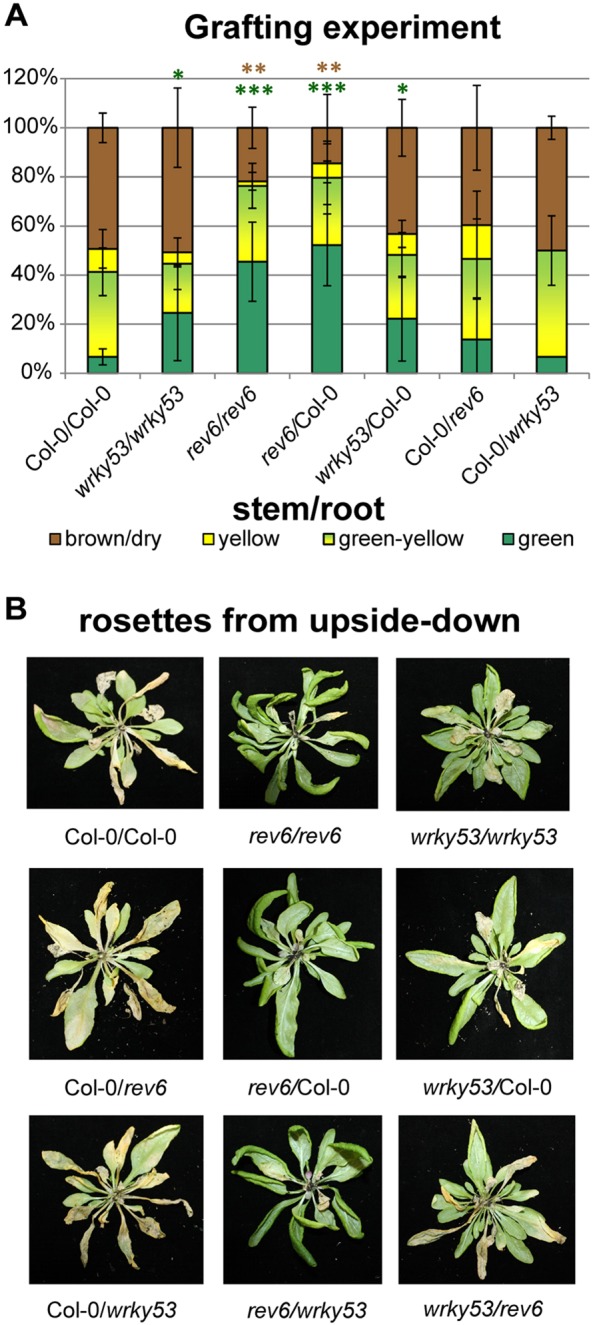
**Grafting experiments and senescence phenotype.** (A) Nine combinations of grafted plants were generated between the wild-type and mutant plants (*rev6* and *wrky53*), including three self-grafted controls, e.g. wild type to wild type (Col-0/Col-0; scion/root). Error bars indicate s.d. (*n*=4-6 independent grafted plants with the exception of Col-0/wrky53, where we achieved only two successful grafts). The quantitative evaluation of leaf senescence of the non-grafted plants is shown in Fig. 5. Asterisks represent significant differences from the Col-0/Col-0 graft, as determined using Student's *t*-test (*P<0.05, **P<0.005, ***P<0.0005). (B) The leaf-senescence phenotypes of grafts. Photographs were taken 7 weeks after grafting.

To further investigate the spatial aspects of *REV* and *WRKY53* expression, we decided to perform grafting experiments with Col-0 wild-type, *rev5* and *wrky53* mutant plants. When the aerial parts of Col-0 were grafted onto either *wrky53* or *rev5* rootstocks, no significant delays in the onset of senescence were observed. However, the converse grafting of the aerial parts of either *wrky53* or *rev5* to Col-0 rootstocks significantly delayed the onset of senescence where the latter again showed a much stronger effect (Fig. 8A,B). The grafting experiments revealed that the root seems not to be involved in the REV/WRKY53-mediated senescence pathway and that depletion of *REV* and *WRKY53* in only aerial tissue strongly affects senescence.

## DISCUSSION

Plants induce leaf senescence to provide carbon, nitrogen and mineral resources to the developing fruits or seeds. Senescence is induced in response to plant age but environmental signals such as light, the availability of water and temperature strongly influence this process. A high-resolution temporal transcript profiling of senescing *Arabidopsis* leaves gives insight into the temporal order of gene activation and repression (Breeze et al., 2011). Approximately 6500 genes are up- or downregulated during the course of leaf senescence, implying an important role for transcription factors in this process. Transcription factors themselves are transcriptionally upregulated in senescing leaves the largest groups being NAC, WRKY, C2H2-type zinc-finger, AP2/EREBP and MYB proteins (Guo and Gan, 2005). Here, we now show that HD-ZIPIII factors, which are known to be involved in basic patterning processes, have an additional role in the latest step of leaf development, the regulation of senescence. REV is a direct and positive regulator of *WRKY53* expression and mutations in *REV* and other *HD-ZIPIII* genes delay the onset of leaf senescence. Interestingly, the delay of the onset of leaf senescence in plants lacking *REV* is stronger compared with plants lacking only *WRKY53*, implying that REV acts also upstream of other senescence-associated genes. In plant lines with even more reduced *HD-ZIPIII* activity, achieved by overexpression of *miRNA165a* (*35S::miR165a*), rosette leaves were so strongly downward curled that it was impossible to determine the onset of senescence. The loss of several *HD-ZIPIII* genes, as in the case of the *phb phv rev* triple mutant, causes severe developmental defects, including consumption of the apical stem cells (Emery et al., 2003; Prigge et al., 2005). The severity of these developmental defects largely precludes a thorough analysis of the general role of HD-ZIPIII proteins at later stages of development. Nevertheless, our findings clearly suggest that the role of HD-ZIPIIIs in promoting senescence is more complex and involves regulation of several senescence-associated target genes. In the *rev5/wrky53* double mutant, leaf yellowing and chlorophyll loss were less severe at later stages than in the *rev* single mutant, whereas senescence-associated gene expression was more severely affected for some senescence-related genes. This clearly points towards a complex network that is altered in different aspects if one or more components are depleted from the system. It was already shown that WRKY53 acts as an upstream regulator, downstream target and protein-interaction partner of WRKY18, which is a negative regulator of leaf senescence, illustrating the complexity of the network and possibly explaining the partially intermediate phenotype of the double mutant (Potschin et al., 2014).

The mechanism by which REV promotes senescence appears to involve transcriptional regulation of direct target genes. Here, we have identified nine genes that are potential direct REV targets that are also differentially expressed during senescence. One of these target genes is *HAT3*, which has been shown to play an important role downstream of REV in the process of setting up polarity in the young leaf primordium (Bou-Torrent et al., 2012; Brandt et al., 2012; Turchi et al., 2013). In young seedlings, *HAT3* expression depends partly on the presence of REV, which is supported by lower levels of *HAT3* mRNA in *rev* mutant seedlings (Brandt et al., 2012). During senescence, *HAT3* mRNA levels decrease with plant age (Fig. 7A). In *rev* mutant seedlings, however, *HAT3* mRNA is more abundant compared with wild type (Fig. 7A). Moreover, the expression levels of several other senescence-related target REV genes changed in a complex way (Fig. 7B-F). These findings suggest that the transcriptome of *rev* mutant plants is profoundly altered, resulting in stage-dependent mis-expression of many differentially expressed senescence-associated genes.

It still remains unclear to which endogenous or exogenous signals HD-ZIPIIIs respond in order to promote senescence. The finding that *WRKY53* expression is strongly upregulated in response to hydrogen peroxide treatment and that this induction is dampened in *hd-zipIII* mutant plants implies that HD-ZIPIIIs might be involved in signal transduction processes in response to changes in the intracellular redox state. Many senescence-associated genes, especially transcription factors of the WRKY and the NAC family, transcriptionally respond to elevated levels of hydrogen peroxide but the mechanism by which the hydrogen peroxide signal is perceived and transmitted is still unclear. Remarkably, the subcellular compartment of hydrogen peroxide production appears to play a role in senescence signaling in which the cytoplasmic H_2_O_2_ is more effective in senescence induction than peroxisomal or mitochondrial H_2_O_2_ (Bieker et al., 2012; Zentgraf et al., 2012). Thus, sensors and mediators of hydrogen peroxide-induced senescence are most likely cytoplasmic and/or nuclear proteins or molecules. During bolting, intracellular hydrogen peroxide levels increase in leaf tissue. This increase is thought to be mediated by a complex regulation of the hydrogen peroxide scavenging enzymes and promotes the onset of senescence (Bieker et al., 2012; Smykowski et al., 2010).

Analysis of the redox sensitivity of the REV protein revealed a reduced DNA-binding ability of REV in response to oxidative conditions, which appears to be a direct effect on the REV protein and does not involve accessory proteins. These results contradict the finding that upregulation of *WRKY53* partially requires HD-ZIPIIIs and indicate a more complex regulatory mechanism. Most likely, DNA-binding of REV is affected by redox changes and also the transactivation activity or protein-protein interfaces, which will be further dissected in the future. However, two of the direct REV target genes encode EAR-domain proteins that are part of transcriptional repressor complexes (Causier et al., 2012). Among these transcriptional repressors are HAT3 and ZFP8, the mRNA levels of which are altered in the senescence process. Therefore, it seems plausible to conclude that REV is a redox-sensitive transcription factor, which among other targets, regulates genes encoding transcriptional repressors. Decreasing REV DNA-binding activity will result in lower expression levels of these transcriptional repressors, alleviating the repressive activity on their targets. Thus, modulation of REV activity in response to alterations of the intracellular redox state will profoundly affect the REV-regulated transcriptome. It is tempting to speculate that also within the shoot apical meristem, domains with different cellular redox states might exist that could serve as positional signals affecting HD-ZIPIII activity.

**Fig. 9.**
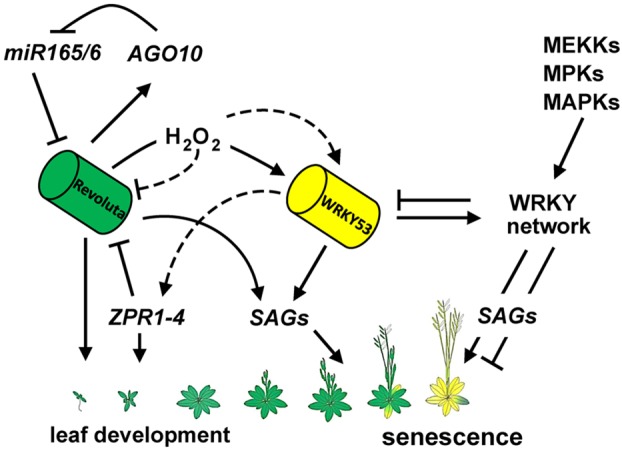
**Model HD-ZIPIII/senescence.** A model summarizing our findings and showing the relationship between early leaf development processes and senescence. Both REV and WRKY53 intersect to regulate the late stages of leaf development.

Developmental age is a major determinant for the induction of leaf senescence in an optimal growth environment. However, when plants are exposed to situations that strongly permit normal growth, senescence is accelerated in order to bypass these adverse conditions and produce seeds that can withstand these adverse conditions. We have tried to depict the complex interplay between REV and WRKY during early and late development in a model (Fig. 9) in which the regulatory cues of REV involving miRNA-dependent regulation through *miR165*, *miR166* and the LITTLE ZIPPER microProteins ZRP1-4 is connected to the MAP kinase-triggered WRKY transcriptional network. Several intersections can be detected between the formerly independently described players in early and late leaf development in which hydrogen peroxide might play a central role.

Shade causes profound developmental changes in shade-sensitive plants aimed at outgrowing competitor plants. We have previously shown that the leaf regulatory module consisting of HD-ZIPIII and KANADI transcription factors is involved in modulating growth in response to shade (Brandt et al., 2012). Consistent with this, shade can also trigger leaf senescence (Brouwer et al., 2012), suggesting that leaf patterning, shade avoidance and leaf senescence are interconnected by differential activity of HD-ZIPIII proteins, thus linking early and late leaf development, and adjusting plant growth and development to changing external conditions.

### Perspectives

It was recently shown that embryonic growth and patterning of mammals largely depends on cellular senescence as a developmental mechanism to shape organ growth (Muñoz-Espín et al., 2013; Storer et al., 2013). This mechanism partly relies on macrophages, which are mobile cells that invade the tissue to remove senescent cells. In this context, senescent cells also produce secreted compounds that can act as positional signals triggering pattern formation and proliferation in adjacent tissue (Storer et al., 2013). The immune system of plants is substantially different from animals and does not involve macrophage-mediated cell clearing. However, it is conceivable that local cellular senescence could provide positional information to direct growth responses. Our finding that HD-ZIPIIIs, which are known basic patterning factors, can influence senescence processes, suggest not only that early and late leaf development are coupled and processes that influence patterning in the early organ control the concerted degradation of tissue during the late phase of development, but also that physiological processes related to senescence, such as nutrient mobilization or lipid peroxidation, might be part of early leaf patterning processes. Furthermore, the puzzling reduction of DNA-binding activity under oxidizing conditions that contradicts the finding that upregulation of *WRKY53* expression by hydrogen peroxide partially requires REVOLUTA prompts us to decipher the redox-dependent changes in the REVOLUTA protein outside the DNA-binding domain in more detail. This, however, will be the subject of further investigations.

## MATERIALS AND METHODS

### Plant material and growth conditions

The following *rev/hd-zipIII* mutant lines were used in this study: *rev-5* (A260V) and *rev-6* (R346STOP), two strong ethyl-methylsulfonate (EMS) alleles (Otsuga et al., 2001), *phb phv rev* triple mutants introgressed in Col-0 (Prigge et al., 2005), *35S::ZPR3* (Wenkel et al., 2007) and *35S::miR165* (Kim et al., 2010). For senescence phenotyping, *Arabidopsis thaliana* plants were grown in a climatic chamber at 20°C under long-day conditions (16 h of light) with only moderate light intensity (60-100 μmol s^−1^ m^−2^) to slow down development for better analyses. Under these conditions, the plants developed bolts and flowers within 5-6 weeks. During growth and development of the leaves, the respective positions within the rosette were color coded with different colored threads, so that even at very late stages of development, individual leaves could be analyzed according to their age. Plants were harvested in a weekly rhythm and samples were always taken at the same time in the morning to avoid circadian effects. For the evaluation of leaf senescence phenotypes, leaves of at least six plants were categorized in four groups according to their leaf color: (1) ‘green’; (2) leaves starting to get yellow from the tip as ‘yellow-green’; (3) completely yellow leaves as ‘yellow’; and (4) dry and/or brown leaves as ‘brown/dry’. Exogenous hydrogen peroxide treatment was conducted by spraying 1%, 0.1% or 0.01% hydrogen peroxide solution including 0.1% Tween20. Grafting experiments were carried out according to Marsch-Martínez et al. (2013).

### Intracellular hydrogen peroxide measurements

After stress treatment, leaf 7 (0.1% H_2_O_2_ treatment) and leaf 8 (heat stress, 2 h at 39°C) were harvested and incubated for exactly 45 min in DCFDA working-solution (2′,7′-dichlorodihydrofluorescein diacetate, 200 µg in 40 ml MS-Medium, pH 5.7-5.8). Leaves were then rinsed with water and frozen in liquid nitrogen. After homogenization on ice, 500 µl 40 mM Tris (pH 7.0) were added and the samples were centrifuged at 4°C for 30 min. Fluorescence (480 nm excitation, 525 nm emission) of the supernatant was measured in a Berthold TriStar LB941 plate reader.

### Chromatin-immunoprecipitation and quantitative PCRs

ChIP and ChIP-qPCRs were carried out as described by Brandt et al. (2012). To quantify gene expression changes, RNA was isolated from seedlings using the roboklon GeneMATRIX universal RNA purification kit following manufacturer's recommendations. One microgram of total RNA was reverse transcribed using the Fermentas RevertAid Premium Reverse transcriptase with oligo-dT primers. cDNAs were diluted 10-fold and 3.5 µl were used for RT-PCR reactions. Quantitative measurements were performed on a Bio-Rad CFX384 using the Fermentas SYBR Green qPCR master mix. Relative quantities were calculated using the delta Ct method and normalized relative to a standard curve. Oligonucleotide sequences are listed in supplementary material Table S1. Further descriptions of the methods can be found in the supplementary material. The ChIP-Seq dataset has been published in the Gene Expression Omnibus database (accession number GSE26722).

### Redox-DPI-ELISA

Recombinant 6xHis-tagged REV protein with and without the PAS domain was expressed in *E. coli* and DNA-protein interaction ELISA was basically performed as described previously (Brand et al., 2010). Crude extracts were pre-incubated with different concentrations of DTT and H_2_O_2_ to examine a redox state-dependent binding of REV (for a detailed description, see methods in the supplementary material).

### Transformation of *Arabidopsis* protoplasts and transient promoter-GUS expression

Protoplasts were derived from a cell culture of *Arabidopsis thaliana* var. Columbia 0 and were transformed with effector and reporter plasmids following roughly the protocol of Negrutiu et al. (1987). The GUS activity assays were carried out as described by Jefferson et al. (1987). A detailed description is presented in the methods in the supplementary material.

### Chlorophyll measurements and phenotypic analysis

For assessment of the leaf senescence state, chlorophyll content of leaf 5 was measured using an atLeaf+ chlorophyll meter (http://www.atleaf.com), lipid peroxidation of leaf 6 was measured using the improved thiobarbituric acid/reactive substances assay, as described previously (Hodges and Forney, 2000), and expression of the senescence-associated marker genes SAG12 (At5g45890) and SAG13 (At2g29350) was analyzed by qRT-PCR. A detailed description is presented in the methods in the supplementary material.

## Supplementary Material

Supplementary Material
