# Supplementary material for: REVOLUTA and WRKY53 connect early and late leaf development in Arabidopsis
Source: Development. 2014 Dec 15;141(24):4772–83. doi: 10.1242/dev.117689 (PMC4299279; doi:10.1242/dev.117689)
Supplement: Supplementary Material [file supp_141_24_4772__index.html]

Supplementary Material 

# REVOLUTA and WRKY53 connect early and late leaf development in *Arabidopsis*

## DEV117689 Supplementary Material

**Files in this Data Supplement:**

- Supplementary Material
