# Supplementary material for: REVOLUTA and WRKY53 connect early and late leaf development in Arabidopsis
Source: Development. 2014 Dec 15;141(24):4772–83. doi: 10.1242/dev.117689 (PMC4299279; doi:10.1242/dev.117689)
Supplement: Supplementary Material [file supp_dev.117689_DEV117689supp.pdf]

Supplementary Material Xie et al.

Supplementary methods

GUS staining and histology. For histochemical GUS staining, *REV::GUS* (*rev-9*) and *WRKY53::GUS* plants were incubated overnight in GUS staining solution (100mM $NaPO_4$ pH7.0, 10mM EDTA, 0.5mM $K_4Fe(CN)_6$, 0.5mM K_3Fe_6, 0.01% Triton-X and 1mM X-Gluc). Samples were cleared with an ethanol series. For histological sections, roots of ten day-old seedlings were stained overnight in GUS staining solution following transfer into fixative (50mM NaPh pH=7.2; 1% Glutaraldehyde; 4% Formaldehyde) for two days. Afterwards, the roots were dehydrated in an ethanol series (30%/ 50%/ 70% each for two hours) and finally stored in 100% ethanol prior embedding in Technovit (Heraeus). Two-micron sections were cut using a Leica microtome.

Redox-DPI-ELISA. The coding sequence of *REV* with and without the PAS domain was cloned into the pET-32b vector for expression of the proteins fused to a 6xHis-tag. The *E. coli* strain BL21-SI was used for protein expression. The cells were grown in 10ml of selective medium overnight and subsequently diluted 1:20 in a final volume of 100ml in medium without antibiotics. Protein expression was induced after 1h by the addition of 1mM IPTG and the cells were grown a further hour at 37°C. After centrifugation (2500g, 20min, 4°C) and washing (10mM Tris-HCl pH 7.5, 100mM NaCl), the bacterial pellet was resuspended in protein extraction buffer (4mM HEPES pH 7.5, 100mM KCl, 8% (v/v) glycerol, 1x complete proteinase inhibitor without EDTA (Roche) and protein extraction was performed by sonication under native conditions. The protein concentration of the crude extract was measured by

Bradford assay (Bio-Rad). Expression of the recombinant proteins was confirmed by Western blot and immune detection using anti-His-antibodies.

The DNA-protein interaction assay was basically performed as described before (Brand et al., 2010). 5' biotinylated complementary oligonucleotides were annealed (final concentration 2M) to get double-stranded DNA fragments. The double-stranded binding sites of REV in the *WRKY53* promoter (BS1 and BS2) were added to a streptavidin-coated ELISA plate (Nunc Immobilizer) for binding for 1h at 37°C. After blocking using blocking reagent (Roche) for 30min, blocking reagent was removed and 25µg crude extracts was added and incubated for 1h at room temperature. Crude extracts were pre-incubated with different concentrations of DTT and H_2O_2 to examine a redox-state dependant binding of REV. In order to test reversibility of the redox-effect, first high concentrations of H_2O_2 were added and subsequently oxidizing conditions were reversed by addition of DTT. After binding, biotinylated DNA-protein complexes were washed two times for 10min at room temperature (blocking solution, Qiagen) and incubated with Anti His-HRP conjugate antibodies (Qiagen) 1:1500 diluted in blocking solution for 1h at room temperature. After washing, interaction was detected by a peroxidase reaction with ortho-phenylenediamine (OPD-tablets, Agilent technologies (Dako)) for 15min in darkness. After stopping the reaction with 0.5M H_2SO_4 solution, positive interactions which resulted in a yellow color could be measured with an ELISA-reader (TECAN Safire XFluor4).

Transformation of *Arabidopsis* Protoplasts and transient Promoter-GUS Expression.

The protoplasts used for the transient expression assay were derived from a cell culture of *Arabidopsis thaliana* var. *Columbia 0*. Cells were transformed with 5 µg of

effector and reporter plasmid DNA each roughly following the protocol of Negrutiu et al. (1987). For details see the following protocol: http://www.zmbp.uni-tuebingen.de/CentralFacilities/transf/index.html. As an internal control 0.1µg of a Luciferase construct was co-transfected. The protoplasts were incubated overnight in the dark and then used for GUS-assays. For the *WRKY53*-reporter, a 2.8kb promoter sequence upstream of the *WRKY53* start codon was cloned into the binary vector pBGWFS7.0 (Karimi et al., 2002) and served as the reporter construct. The *REVOLUTAd* CDS in the pJAN33 vector (Weigel et al., 2003) served as an effector. The GUS activity assays were carried out as described by Jefferson (Jefferson et al., 1987). To normalize differences in protoplast transfection a luciferase-assay was performed using the Dual-Luciferase® Reporter Assay System from Promega following the user´s manual (Promega,http://www.promega.de/~/media/Files/Resources/Protocols/Technical%20Bulletins/0/Luciferase%20Assay%20System%20Protocol.pdf). In total 6 biological replicates with 3 technical replicates each were performed for the experiment.

Chlorophyll measurements and phenotypic analysis. For assessment of the leaf senescence state, color-coded leaves were used. Chlorophyll content of leaf No. 5 was measured using an atLeaf+ chlorophyll meter (http://www.atleaf.com). Total chlorophyll content of leaves can be obtained by converting the atLEAF+ values in SPAD and considering the relation among chlorophyll content and SPAD units (http://www.atleaf.com). Each leaf was measured in triplicate at three different positions. Chlorophyll was determined for three independent plants and values were averaged. In addition, expression of the senescence associated marker genes *SAG12* (At5g45890) encoding a cysteine protease and SAG13 (At2g29350) encoding a short-

chain alcohol dehydrogenase was analyzed by qRT-PCR and normalized to the expression of the GAPDH gene (At1g13440). Lipid peroxidation was measured using the improved thiobarbituric acid-reactive-substances assay as described previously (Hodges et al., 1999). Leaf No. 6 of three different plants was homogenized in liquid nitrogen and 25 mg of the fine leaf tissue powder was used for analyzes.

Supplementary figures

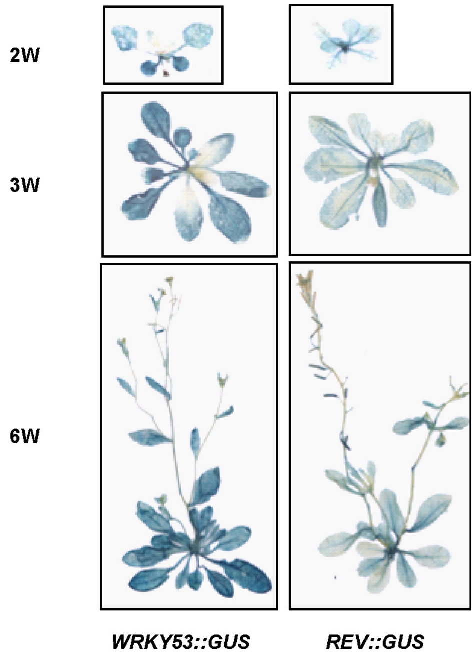

Suppl Fig. S1. Expression analysis of *REV* and *WRKY53*. Spatial expression patters of *REV* and *WRKY53* using histochemical staining of 2-week to 6-week-old rosettes of p*WRKY53*::GUS and p*REV*::GUS plant lines.

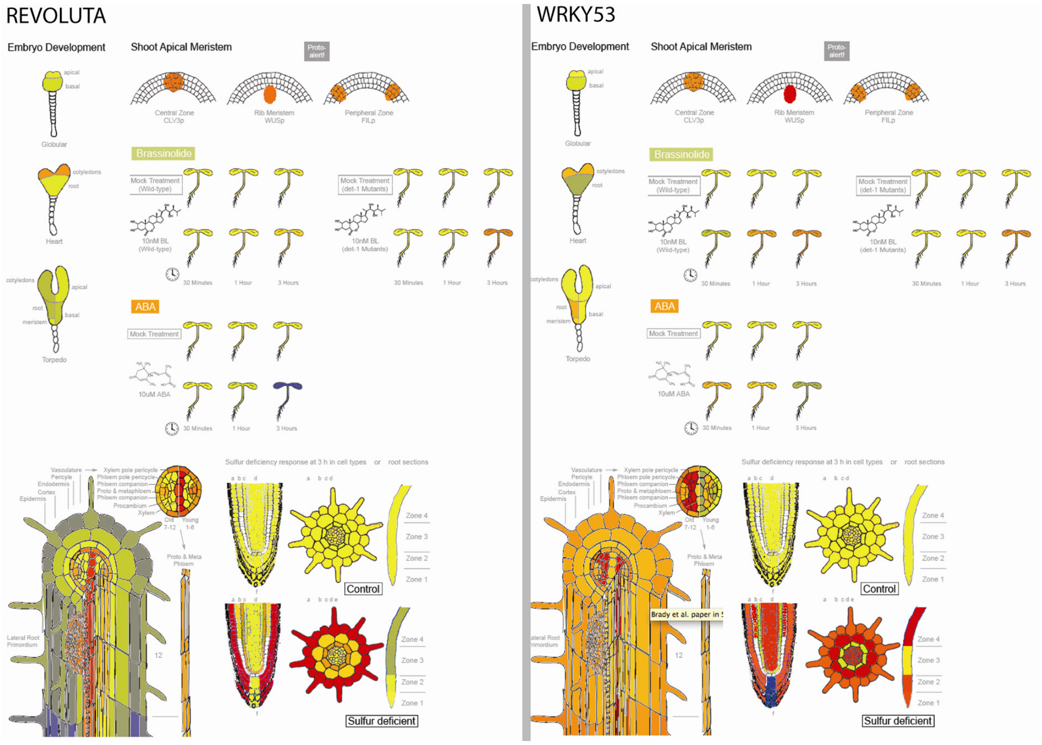

Suppl Fig. S2. Publicly available microarray-based expression analysis using Arabidopsis eFP browser showing co-expression of *REV* and *WRKY53* in different tissues. Plotted are images showing relative expression values where red color indicates high expression and blue color indicates low expression. Intermediated expression levels are displayed in yellow and variations between yellow and red and yellow and blue.

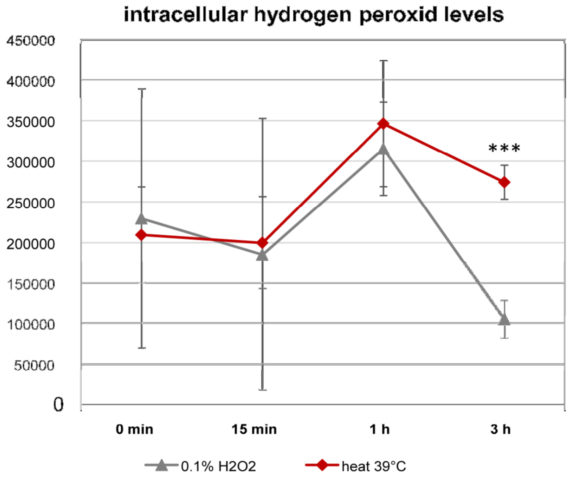

Suppl. Fig. S3. Measurement of intracellular H_2O_2 contents using DCFDA after 01.% H_2O_2 application or heat treatment. Error bars indicate standard deviation of at least three biological replicates, *** p<0.0005 (T-Test).

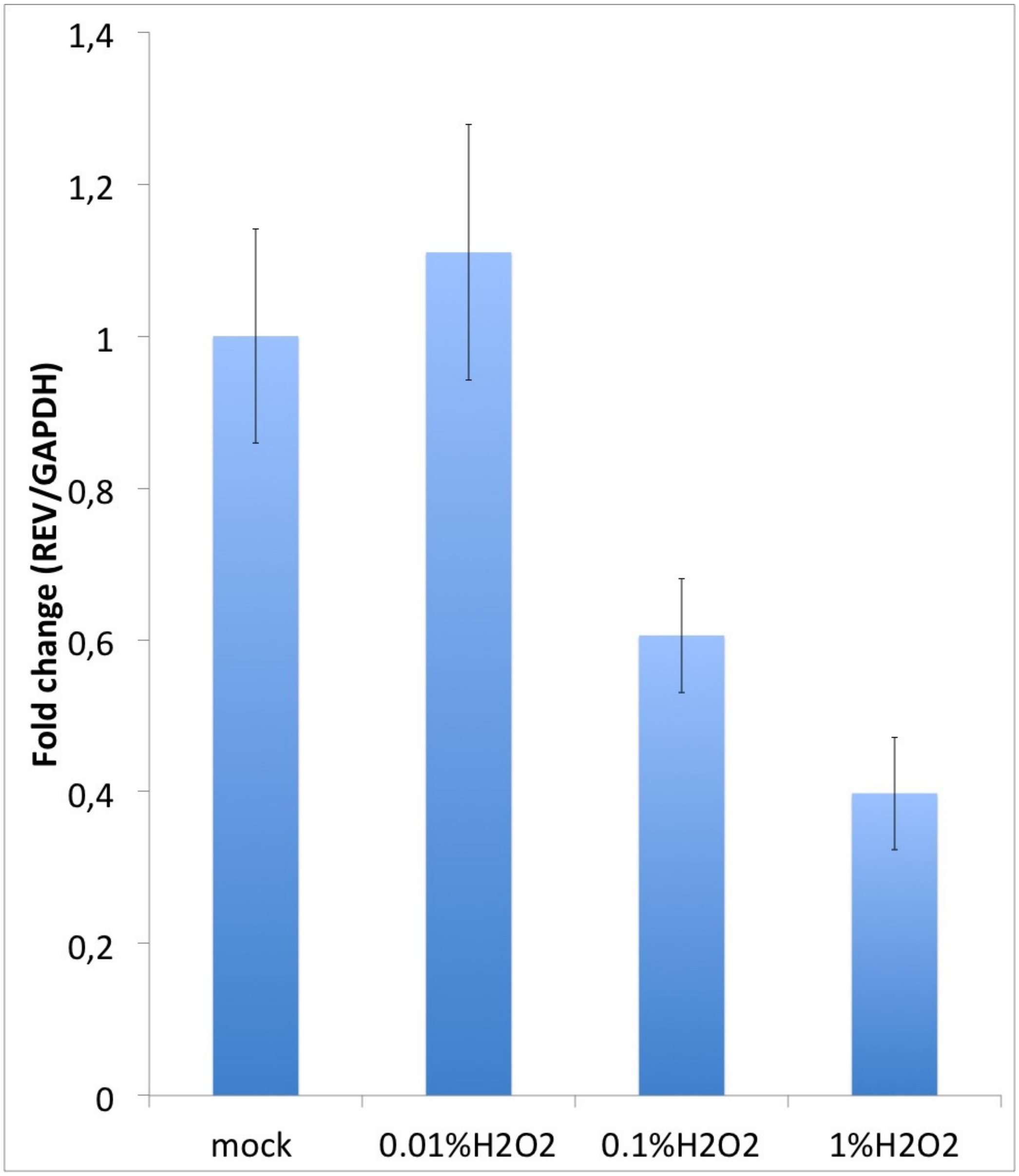

Suppl. Fig. S4. Analysis of REV mRNA changes in response to hydrogen peroxide. Real-time qPCR experiment showing *REV* expression in response to hydrogen peroxide treatment in wild type. 3-week old plants were treated with different concentrations of H_2O_2 (0%, 0.01%, 0.1% and 1%) for 40 min. Plotted are representative relative expression changes (fold change) of the mean of four technical replicates including standard deviations. Similar expression changes have been observed in at least two independent biological experiments.

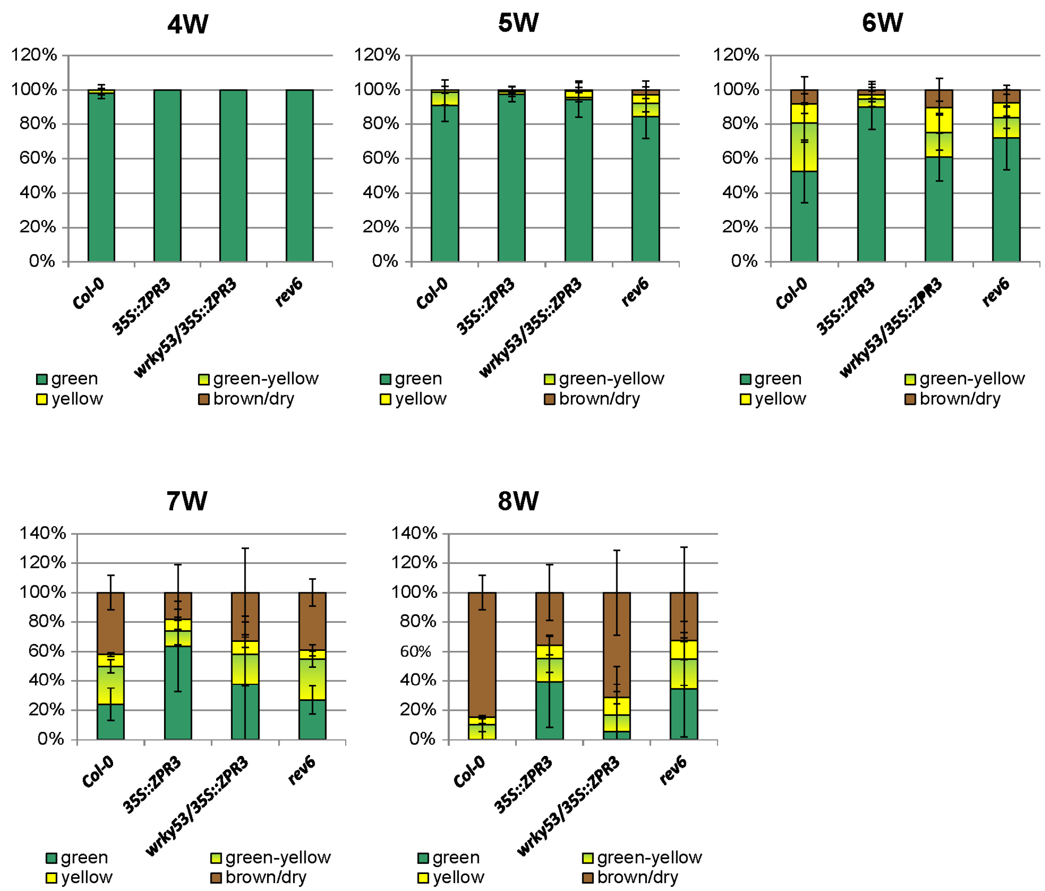

Suppl. Fig. S5. Quantitative evaluation of senescence in additional lines with altered HD-ZIPIII expression.

For a quantitative evaluation of leaf senescence plants were harvested in a weekly rhythm and leaves of at least ten plants were categorized into four groups according to their leaf color: 1) “green”, 2) leaves starting to get yellow from the tip as “yellow-green”, 3) completely yellow leaves as “yellow” and 4) dry and/or brown leaves as “brown/dry”. The percentages of each group with respect to total leaf numbers are presented. Error bars indicate standard deviations. Col-0 (wild type); *rev6* (different

rev mutant line); *35S::ZPR3* (plant line overexpressing the small leucine-zipper-type microProtein ZPR3, a micro protein inhibiting HD-ZIPIII function by protein-protein interaction); *wrky53/35S::ZPR3* (plant line overexpressing *ZPR3* in the *wrky53* mutant background).

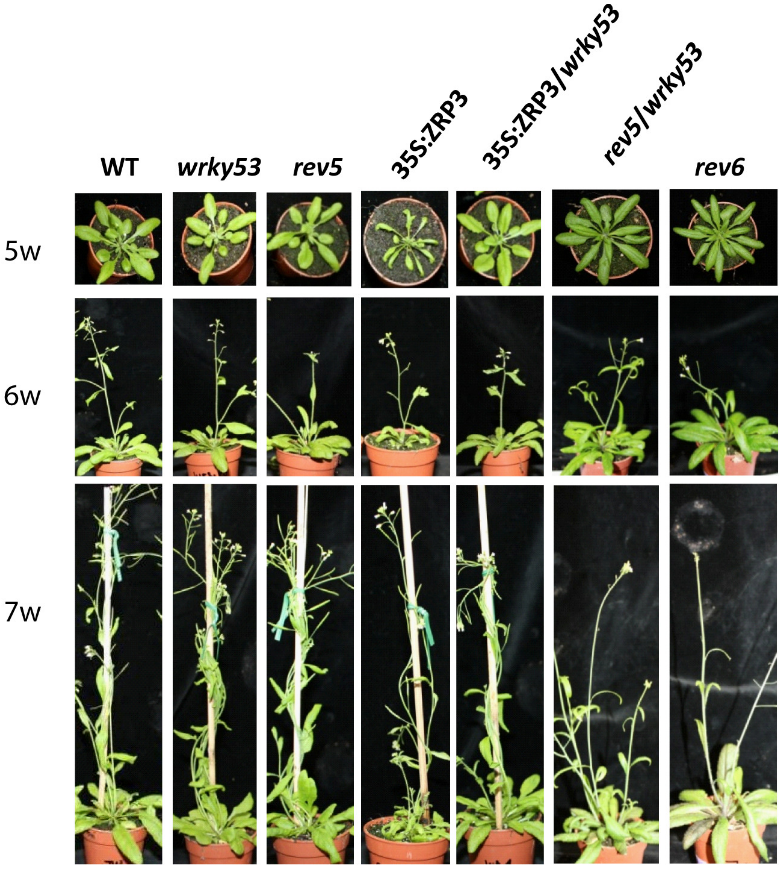

Suppl. Fig. S6. Comparison of plant development of WT, *wrky53*, *rev5*, *wrky53/rev5*, *35S::ZRP3* and *35S::ZPR3/wrky53* and *rev6* plants from 5-week to 7-week-old plants

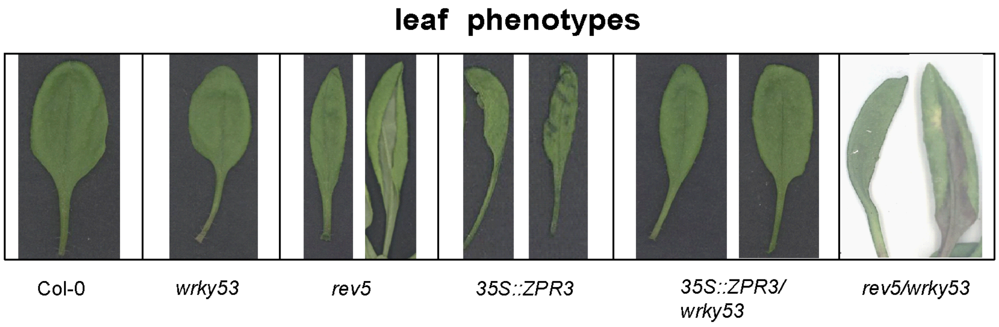

Suppl. Fig S7. Phenotype of leaves of 6-week-old plants of different plant lines.

Table 1. Oligonucleotides

Primers	Sequence	Use
W53CpAf	tgctcaagtttctgggaaaaa	ChIP-qPCR (Primer I)
W53CpAr	aagacgtgctgtcctctgaa	ChIP-qPCR (Primer I)
W53CpBf	caatgaatgaatgacgcaaaa	ChIP-qPCR (Primer II)
W53CpBr	ttcaaaaggacaggattgga	ChIP-qPCR (Primer II)
W53CpCf	cgtatcacacagtgactggttttt	ChIP-qPCR (Primer III)
W53CpCr	ctgaccaagtcaccatggaa	ChIP-qPCR (Primer III)
qW53f	aaactgttgggcaacgaaac	ChIP-qPCR (Primer IV)
qW53r	aatggctggtttgactctgg	ChIP-qPCR (Primer IV)
REVe56F	tttctttgcctaattgcttgg	rev5 genotyping (with rev5R and BaeI digest)
rev5R	tcataaaggggtcgaagcac	rev5 genotyping (with REVe56F and BaeI digest)
vam-F_rev6	tgctgcttcagcttctcagt	rev6 genotyping (with REV-3R and TaqI digest)
REV-3R	gaagcacctccaaccgttaga	rev6 genotyping (with vam-F_rev6 and TaqI digest)
W53tDNAr	gttcaagtcctgttgaacattcc	wrky53 genotyping
W53tDNAf	ggcagtgttccagaatctcc	wrky53 genotyping
LB-Salk	tggttcacgtagtgggccatcg	wrky53 genotyping
AtREV-topo_F	caccaacatacatgacatgtgaaat	Cloning of the pREV::REV::GFP construct
AtREV-topo_R	cacaaaagaccagtttacaaaggag	Cloning of the pREV::REV::GFP construct
qGAPDH_F	aaagtgttgccatccctcaa	AT5G47180 qRT-qPCR
qGAPDH_R	tcggtagacacaacatcatcct	AT5G47180 qRT-qPCR
qWRKY53_F	ggcagtgttccagaatctcc	WRKY53 qRT-PCR
qWRKY53_R	gcctctctctgggcttattc	WRKY53 qRT-PCR
qSAG12_F	ggaggaaaacaatcgctacg	SAG12 RT-qPCR
qSAG12_R	acggcgacattttagtttgg	SAG12 RT-qPCR
qSAG13_F	gtgccagagacgaaactc	SAG13 RT-qPCR
qSAG13_R	gctgtaaactctgtggtc	SAG13T-qPCR
qHAT3_2f	ggagtttcgtctccgaacag	HAT3 RT-qPCR
qHAT3_2r	atcggagtttcttcctcgaa	HAT3 RT-qPCR
qZFP8_F	ccgccattattcgtctcttc	ZFP8 RT-qPCR
qZFP8_F	gatgtccacctagggcttga	ZFP8 RT-qPCR
qIDD11_F	tggcaacaaacagattcgtg	IDD11 RT-qPCR
qIDD11_R	gatggatcatggtggacaca	IDD11 RT-qPCR
qAT5G47180_F	caagcgcaacgagagtatcc	ZFP8 RT-qPCR
qAT5G47180_R	ttgggttgtagacggggtaa	ZFP8 RT-qPCR
qAT1G74940_F	ggtattgttgctgcgttgga	AT1G74940 qRT-qPCR
qAT1G74940_R	acaattcaaacccgtcgtcg	AT1G74940 qRT-qPCR
qAT1G49200_F	tctgttgcctcgggttacat	AT1G49200 qRT-qPCR
qAT1G49200_R	cggcaagttcatctcaggtg	AT1G49200 qRT-qPCR

Supplementary references

Karimi, M., Inze, D. and Depicker, A. (2002). GATEWAY vectors for Agrobacterium-mediated plant transformation. Trends Plant Sci 7, 193-195.

Weigel, M., Varotto, C., Pesaresi, P., Finazzi, G., Rappaport, F., Salamini, F. and Leister, D. (2003). Plastocyanin Is Indispensable for Photosynthetic Electron Flow in Arabidopsis thaliana. Journal of Biological Chemistry 278, 31286-31289.
